# Fiddler crab claws work as a deflection antipredator defence

**DOI:** 10.1098/rsbl.2024.0694

**Published:** 2025-06-18

**Authors:** Diogo Jackson Aquino Silva, Samuel Bear Powell, Marilia Fernandes Erickson, Fabio Cortesi, Daniel Marques Almeida Pessoa, Karen Louise Cheney

**Affiliations:** ^1^ Department of Physiology and Behavior, Universidade Federal do Rio Grande do Norte, Natal, Rio Grande do Norte, Brazil; ^2^ School of the Environment, The University of Queensland, Brisbane, Queensland, Australia; ^3^ Queensland Brain Institute, The University of Queensland, Brisbane, Queensland, Australia; ^4^ School of Natural Sciences, Macquarie University, Sydney, New South Wales, Australia

**Keywords:** *Gelasimus vomeris*, predation, body coloration, autotomy, decoy, predator–prey interaction

## Abstract

Conspicuous coloration in body parts that can be autotomized, diverting predator attacks from vital to non-vital regions, is called deflection. Fiddler crabs typically have a cryptic or conspicuous carapace (vital area), while the claw (non-vital) is often conspicuous and used for social communication. Here, we tested whether the conspicuous claws of fiddler crabs divert predator attacks away from their carapaces, enhancing survival. To do this, we used a robotic crab model that replicated the colours and reproductive waving display of the two-toned fiddler crab, *Gelasimus vomeris*. Models were placed in the field to be attacked by Australian brush-turkeys, *Alectura lathami*. We analysed whether the first attack was directed at the claw or the carapace with differently coloured models. Our results show that robot crab models with conspicuous claws drew half of the attacks to the claw, whereas models with non-conspicuous claws were attacked predominately on the carapace. This suggests that the claws of the fiddler crabs effectively attract attacks away from the carapace, functioning as a deflection mechanism. This is the first study demonstrating a claw-deflection strategy in crustaceans, indicating that the claw not only plays a role in intraspecific signalling but also mitigates associated predation risks.

## 1. Introduction

Animal body coloration may play an important role in signalling during reproductive interactions [1–3] and in prey–predator interactions [4,5]. Antipredator strategies may include cryptic coloration for camouflage [6], or conspicuous coloration to advertise defensive capabilities [4]. Additionally, conspicuous coloration, often paired with other behaviours, can confuse predators through mechanisms such as flash displays [7,8], deimatic displays [9] or deflective coloration [10].

Deflection defence uses coloration to influence a predator’s initial attack site, diverting attacks to a body region that is difficult to grasp or that can be autotomized or broken off, increasing the preys' chance of survival [10]. Body coloration is effective in deflecting predator attacks, as demonstrated by colourful tails in lizards [11–14] and colourful wing tails in butterflies [15]. Deflection is suggested as an antipredator defence for multiple other taxa, such as in many insects, tadpoles and weasels [10], but it has not been empirically tested in most cases [5]. Recently, it has been suggested that crustaceans, especially fiddler crabs, may use the conspicuous claw as a deflection mechanism to reduce the cost of predation associated with waving displays [16].

Fiddler crabs are sexually dimorphic, with males possessing a hypertrophied claw, which plays an important role in both agonistic and antagonistic social interactions [17,18]. Traits such as claw size [17], waving rate [19–21] and coloration [2,16,22–24] mediate these social interactions. While the claw colour exhibits a stable and conspicuous signal, the carapace is variable, adjusting its appearance over the course of a year (at the population level) or within just a few minutes (under stressful conditions) [25]. This suggests that the claw colour is always conspicuous and ready for signalling, whereas the carapace can become conspicuous for signalling during the reproductive period [24,25] or for thermoregulation [26] or else remain camouflaged. The claw’s colour and movement, while primarily signalling to conspecifics, can also make the crabs more detectable to predators [16,27–29].

To mitigate the survival costs associated with signalling, fiddler crabs employ behavioural survival tactics such as fleeing (30), pinching, raising and moving the claw [31] using it as a shield, and when captured, can autotomize legs and claws [32,33]. They can also autotomize the claw after pinching, potentially giving them time to escape. Although waving displays are not intended to attract predators, males can still be attacked by surprise while displaying to females. Furthermore, predation pressure on crabs can drive risk-reducing strategies such as camouflage [28,34,35], and aposematism [34,36]. Despite this, deflection as a potential defence mechanism remains untested.

In this paper, we examined whether the hypertrophied claw of two-toned fiddler crab (*Gelasimus vomeris*) functions without diverting predator attention away from the carapace, characterizing this as deflection. Using a robot crab model, we exposed birds to combinations of conspicuous and cryptic carapace and claw colours (orange claw and black carapace; orange claw and blue carapace; and black claw and black carapace) and recorded their attack pattern. We hypothesized that fiddler crab claw coloration would work as a deflection by altering the predator’s initial attack. Specifically, we predicted that the orange claw, due to its conspicuousness (movement and colour), would deflect attacks from the carapace.

## 2. Methods

### (a) Study site

The study was conducted in Brisbane, Queensland, Australia, in 12 different urban locations, including parks and areas with bushes, and where Australian brush-turkey (*Alectura lathami*), our predator model, was present (specific locations are shown in electronic supplementary material, table S1). The experiment involved positioning the fiddler crab robot models to observe attacks by brush-turkeys. The samples were collected on sunny or partly cloudy days, between 09.00 and 16.00, from April to June 2024.

### (b) Experiments

Our initial aim was to conduct the study in mangroves and parks with the presence of the Australian white ibis, a natural predator; however, we were unsuccessful. Therefore, we used the Australian brush-turkey as a proxy, as it responded well to our model. Although brush-turkeys are not natural predators of fiddler crabs, they are a suitable proxy for a predator due to their behaviour of foraging on the ground, which is similar to natural predators like the white ibis [31]. We presume they have a violet-sensitive tetrachromatic vision (order Galliformes), which is the same as shorebirds that predate on crabs (e.g. terns) (order Charadriiformes) [37].

The experiment was conducted using a single robotic model that was positioned in areas where Australian brush-turkeys were foraging in urban areas far from any live fiddler crabs. The robotic fiddler crab was activated at a distance of 5 m to perform the up-down waving motion (comprised of 1 s of motion followed by 1 s of pause) (electronic supplementary material, video S1) until it was attacked by a brush-turkey (electronic supplementary material, video S2). After positioning the model, the range of time for the bird to attack was between 1 and 30 min. During the attack, the following data were registered: (i) the location of the first attack on the model (i.e. claw or carapace), (ii) the type of illuminant (i.e. shade or direct sunlight), (iii) the attack position relative to the model (i.e. frontal, from the back, or lateral), and (iv) the collection site coordinates (electronic supplementary material, table S1). Each brush-turkey was used only once to avoid repeated sampling. To ensure this, data collection followed a fixed directional path at each site using always the same treatment in a given location. Additionally, sites were located at least 1 km away from prior locations. Some locations (i.e. The University of Queensland, Botanic Gardens at Mt Coot-Tha, Captain Burke Park and Simpson Playground Reserve) were revisited after two weeks for a different treatment, potentially involving the same brush-turkeys. With the repetition, a single individual may have potentially attacked up to two models with different treatments; however, these trials were accounted for in the statistical model. After each attack, the model was inspected for damage to its movement mechanisms or paint. If the claw or carapace was damaged, it was replaced with an identical one.

**Figure 1.**
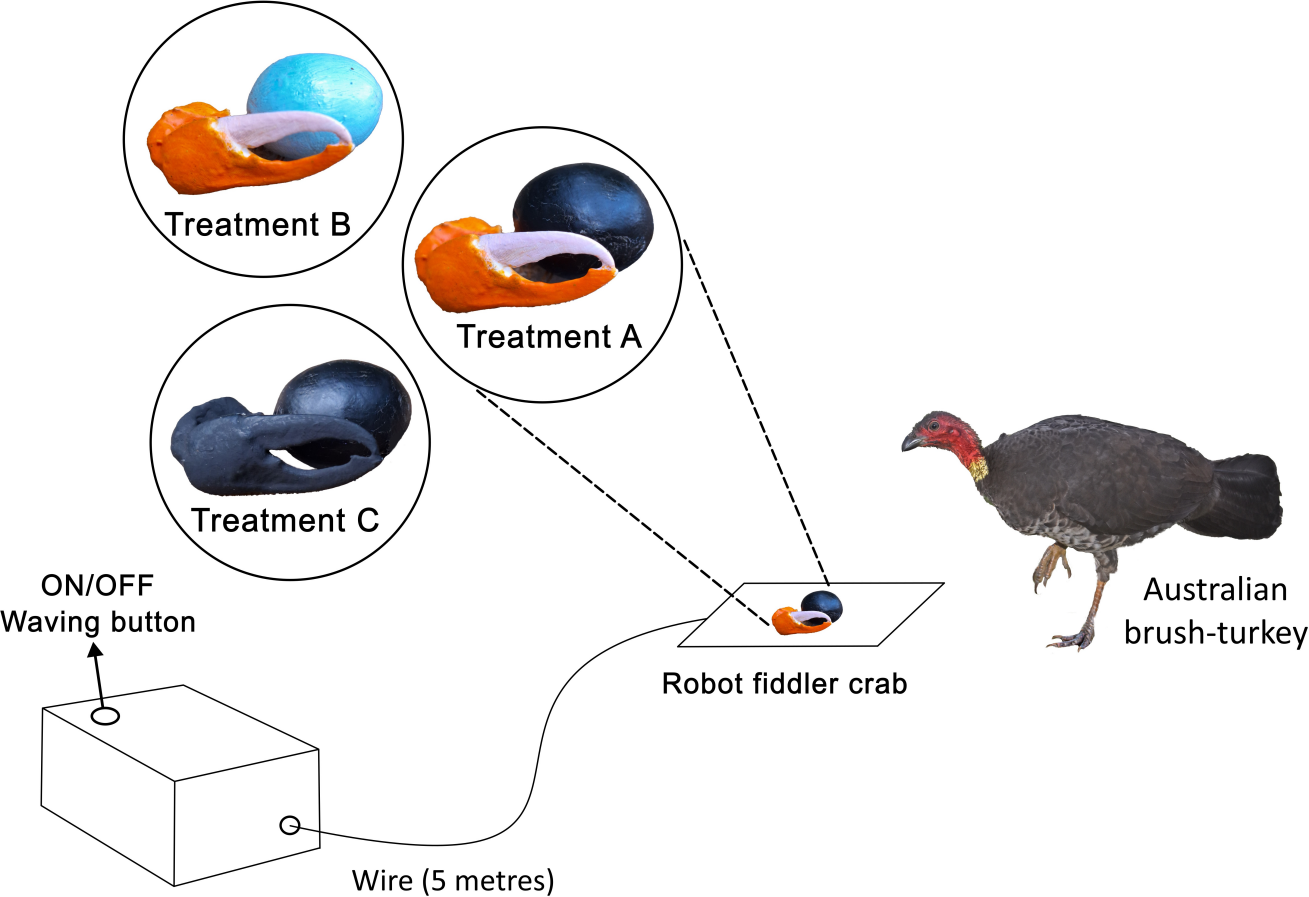
Experimental design and colour treatments. The robotic fiddler crab was positioned on top of a mud-coloured square piece of cloth, and its waving motion was activated/deactivated by a button on the mechanism contained within the box. A steel cable connected to the servo motor in the box moved the robot’s claw from 5 m. We tested three colour treatments of the robotic crab: (A) orange claw, black carapace, (B) orange claw, blue carapace and (C) black claw, black carapace.

Three colour treatments were used as robot crab models: (A) orange claw and black carapace (*n* = 32 attacks), (B) orange claw and blue carapace (*n* = 31) and (C) black claw and black carapace (*n* = 28) (figure 1). Treatments (A) and (B) represent the natural coloration of the fiddler crab, while treatment (C) represents a control hypothetical fiddler crab that does not present any coloration capable of deflecting predator attacks. We attempted to collect data on a fourth treatment (i.e. treatment D) with both claw and carapace painted black and without the waving movement, but the brush-turkeys largely ignored it. All treatments used robots of identical size, shape, movements and waving speed, differing only in colour.

### (c) Robot fiddler crab

The robotic model of a fiddler crab was made with a plasticine carapace (with a diameter of 2 cm) and a movable claw (3 cm in length) that simulates the waving display of the fiddler crab. The characteristics of the claw (i.e. size, shape, colour and waving) were replicated based on the features of an adult male individual of the two-toned fiddler crab, *G. vomeris*. The carapace of the robotic models was simplified and did not include legs or eyes, but the coloration was designed to mimic the natural colour of *G. vomeris*. We did not control for the reflectance of polarized light that these animals may exhibit, as birds cannot perceive polarization [38,39].

To create the model, we scanned an autotomized fiddler crab’s claw (*G. vomeris*) with a three-dimensional scanner (EinScan Pro HD, Shining 3D), creating a three-dimensional digital model of the claw’s size and shape (see robotic crab’s electronic supplementary material). This model was used to print a three-dimensional replica of the claw using a three-dimensional printer with biodegradable polylactic acid (PLA) material. The coloration of the claw (manus and dactylus) and carapace of four animals were measured in the field. The reflectance spectra of one of them (an individual that simultaneously exhibits patches of both black and blue on its carapace) were then used as a reference to paint the models.

The fiddler crab *G. vomeris* exhibits different patterns on its carapace, which can vary from completely black to fully light blue carapaces, including some blueish-white spots or patterns between these two [28]. However, to avoid the influence of spots and patterns and standardize the treatments, we focused on the extreme phenotypes (fully black and fully light blue) paired with a conspicuous orange claw.

**Figure 2.**
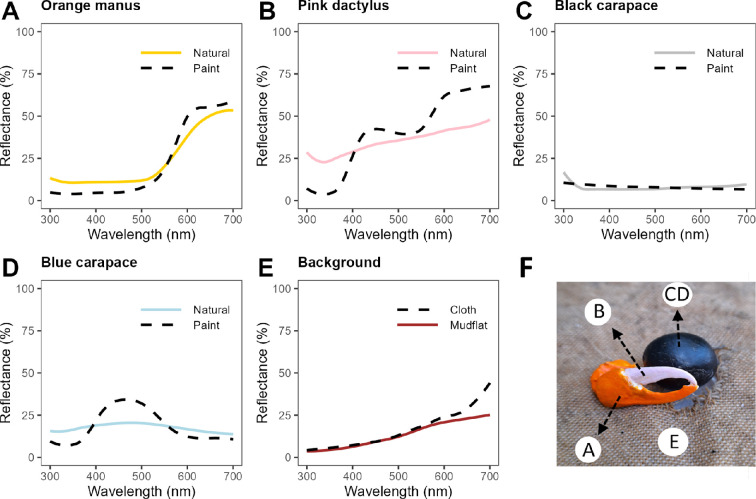
Comparison of the spectral reflectance from two-toned fiddler crab, *Gelasimus vomeris*, the background it is found on (i.e. mudflat), and the robotic model (A–E). Crab model with labels of location of measured spectra (F). Solid lines denote real crab measurements, dashed lines are the replicated colours.

The reflectance of both live and robot crabs was measured using a USB4000 spectrometer (Ocean Optics, Dunedin, FL, USA) and a 400 um fibre optic cable (Thorlabs, NJ, USA), using the sunlight as the light source and the probe positioned at a 45° angle. We used a white standard (WS−1-SL Spectralon) for the full-illumination reflectance standard and blocked the light with black velvet cloth to obtain a dark reference. To replicate the colours of the fiddler crab *G. vomeris*, we compared both the visual appearance and the reflectance spectra. The paint mixtures were prepared using white, black, red, yellow and blue Anko acrylic paints (Anko Global Holdings Pty Ltd, Victoria, AUS) and orange Kaiser craft acrylic paint (KC306). We produced orange (1 : 1 orange to yellow paint), desaturated pink (1 : 20 red to white paint) and light blue colours (1 : 20 blue to white) with a relatively similar reflectance to the natural colours (figure 2).

The waving display of the robotic model (electronic supplementary material, video S1) imitated the wave of *G. vomeris* based on *in situ* observations (electronic supplementary material, video S3). These crabs use their hypertrophied claw to perform the stereotypical up-down movement [40]. To mimic this movement, we used a servomotor controlled by a Pololu Micro Maestro six-channel USB Servo Controller programmed to perform a one-second wave motion every 2 s (1 s to perform the movement, followed by a 1 s pause before the next movement) (see robotic crab’s electronic supplementary material to access the code). The motor, controller and battery (NXE Power 14.8 V, 1300 mAh Li-Po) were housed inside a waterproof box and connected to the claw replica via a 5 m steel Bowden cable (figure 1). The claw replica and carapace were fixed to a wooden base covered with a 40 cm² cloth, leaving only the claw replica and carapace replica visible. This cloth was soiled with mangrove substrate and kept wet during the experiment to simulate the colour of the real substrate, ensuring the model’s contrast with the substrate was controlled, even when placed in different environments with different backgrounds (e.g. grass, concrete, sand).

### (d) Statistics

To assess the influence of coloration on bird attacks, we used a generalized linear mixed model (GLMM) via the g*lmer*() function with a binomial distribution from the ‘lme4’ package [41] in the R program [42]. In this model, the binomial variable ‘first attack’ (claw or carapace) was the response variable. We included the fixed predictor variables ‘colour treatment’ (A, B or C; figure 1), ‘attack position’ (attacks from the back, front or lateral), and ‘illuminant’ (direct sunlight or shade). We included random effects 'trial' and 'location' to account for variability across experimental set-ups between trials (e.g. different times of day) and locations (e.g. habitat characteristics, brush-turkey identity). Since the attack position and illuminant did not show a significant effect (electronic supplementary material, table S2), and based on the likelihood ratio test (LRT), which showed no significant loss of model fit (*p* = 0.5141) after their removal, these variables were excluded from the final model.

## 3. Results

**Figure 3.**
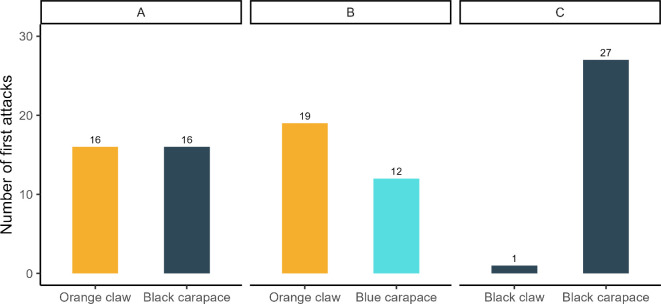
Total number of first attacks on claws and carapaces in treatments A (orange claw and black carapace), B (orange claw and blue carapace) and C (both claw and carapace black).

For the natural coloration treatments A (orange claw and black carapace) and B (orange claw and blue carapace), the claw received 50% (16/32) and 61.2% (19/31) of attacks, respectively. In contrast, our results show that for treatment C (black claw and black carapace), the carapace received 96.3% of the first attacks (27/28), significantly reducing attacks on the claw when compared to the reference factor treatment A (estimate = −3.296, SE = 1.078, z = −3.057, *p* = 0.002) (figure 3, electronic supplementary material, table S2). In treatment D (which is treatment C, but without the waving movement of the claw), the brush-turkeys were not attracted to the model and tended to ignore it.

## 4. Discussion

This is the first study to demonstrate that the coloration of a fiddler crab’s claw may deflect predators away from the carapace. Our results show that the number of attacks on the claw and carapace is similar when replicating the real coloration of fiddler crabs (i.e. treatments A and B). However, in our control treatment (C), in which the claw is black like the carapace, attacks on the carapace are significantly higher. Considering that the carapace has a spherical shape, this may have exploited an innate preference of brush-turkeys for small fruits or seeds [43]. Similar to in chicks, where stimulus shape changes the innate preference of same coloured stimulus and prey movement does not affect chick preference [44]. Here, when the claw did not exhibit conspicuous coloration, they instead preferred to attack the more attractively shaped (i.e. spherical carapace) part of the robot crab. This indicates that the orange colour of the claw may be essential in the predation sequence and that at least 50% of the time (when the carapace is black) and 61% (when the carapace is blue), the claw functions by deflecting attacks from the carapace. This suggests that the waving display of the fiddler crabs strongly attracts the predator’s attention, but when the claw is orange, part of that attention is directed specifically to the claw. Outside the reproductive period, waving is likely not an advantageous strategy, as in this case, remaining unnoticed might be more important than relying on deflection.

The brush-turkeys attacked the model only once or twice, but this does not necessarily mean they were not interested in the model itself, but rather that they were able to recognize it was not food after the first attack. As Bateman *et al*. [45] suggest, birds are capable of learning and avoiding models that do not provide any reward. Other bird species, however, could respond differently.

The coloration of fiddler crabs typically consists of a conspicuous claw that stands out from the rest of the body and the substrate when seen by an avian predator [16,27]. Many fiddler crab species have the ability to modify the coloration of their carapace, becoming lighter or darker depending on the temperature [26], reproductive period [18,25] and predation pressure [28]. In an environment with high predation pressure, *G. vomeris* changes the light blue coloration patterns of its carapace to become darker [28].

Unlike live crabs, our robot does not have the ability to respond to predators. The consequence of this is that the brush-turkeys were able to choose when to attack and from which position to attack. In a realistic scenario, fiddler crabs do not actively offer their claw to predators, but males can become vulnerable to surprise attacks during the waving display. In such cases, the claw may divert the predator’s attention and help prevent a fatal strike to the carapace. When not caught by surprise, fiddler crabs may flee to their burrows or, if they do not have one, defend themselves by using their claw as a shield, raising it in front of their body [31]. This could additionally induce predators to attack the claw and might add the step of claw autotomy to the sequence of anti-predator strategies used by fiddler crabs, which increases predator handling time [46]. Despite our experimental limitation, the crab’s behavioural response could potentially further complicate the predation attempt. Future studies with live crabs or field data could offer more insights into how behaviour affects predation sequence and the efficacy of deflection.

We have shown that brush-turkeys will divert half of their attacks when the claw is a different colour than the carapace. When a predator attacks a lizard that makes use of deflection as an anti-predator strategy, it can still eat the autotomized tail, which means that the interaction still provides benefits to the predator [47]. We are not aware that autotomized claws are a source of nutrition for predators. In personal observations (D.J.A.S.), claws were never eaten when the crab was predated on, and autotomized claws can be found on the mudflat near bird footprints. If claws are not consumed by predators, and there is a negative correlation between size and pinch force in fiddler crab’s claws [48], the claw may not signal a danger to predators, according to the weakening combatant hypothesis [49], but rather unprofitability due to deflection. Thus, it is possible that after several unsuccessful attempts to capture a crab with a conspicuous claw post-autotomy, predators may learn to avoid males with such claws. Indeed, ibises prefer to prey on females or males without a claw rather than on males with hypertrophied claws [46]. We suggest that aposematism (unprofitable) can also complement deflection by increasing the time predators spend foraging to unsustainable levels. This is similar to evasive mimicry, where predators learn to avoid prey that they cannot catch [50].

Studies on lizards indicate that their tails serve as decoys, attracting predator attacks and deflecting attention away from vulnerable areas like the thorax or head, particularly when the tail displays conspicuous colouring [11,14,47]. We show here that the brightly coloured claws of fiddler crabs work in a similar way, by drawing predator attention away from the main body. Both structures also share other similarities. For example, if attacked, both can autotomize, maintaining reflexive movements to distract the predator while the prey escapes. Coloration patterns to deflect predator attacks are also important in butterflies, where the combination of wing extension and colours attracted more attacks than the rest of the body and wing [15]. It remains to be tested if the fiddler crab claws with distinct patterns are better at deflecting predators compared to the plain-coloured phenotypes.

Fiddler crab claws likely evolved primarily as signalling structures. However, occasional predator attacks directed at the conspicuous claw may have been positively selected, as this could increase the individual’s chances of survival. In this context, other crabs that use their claws for signalling may exhibit similar strategies, such as ghost crabs (Genus *Ocypode*) and semaphore crabs (*Heloecius cordiformis*). In addition to crabs, other decapod species such as the giant river prawn (*Macrobrachium rosenbergii*), which have brightly coloured claws, are suggested to use deflection as a defence mechanism [51]. Our results open up a range of possibilities for future studies to explore new antipredator strategies in crabs and other crustaceans.

In conclusion, we demonstrate that the coloration of fiddler crab claws can attract predator attention, thereby reducing fatal attacks to the carapace, similar to the function of colourful lizard tails. Future studies should focus on analysing the behaviour of natural predators, examining how the predator’s experience affects its decision-making, and investigating how multiple defensive strategies work together to increase the survival rate of fiddler crabs.

## Data Availability

All data and R codes are available via the Zenodo Digital Repository [52]. The electronic supplementary material for the robotic crab, including the operating code, three-dimensional models for printing, videos demonstrating its functionality and electronic supplementary material, tables 1 and 2, is available from the Dryad Digital Repository [53].
